# Comparative Analysis of the Placental Microbiome in Pregnancies with Late Fetal Growth Restriction versus Physiological Pregnancies

**DOI:** 10.3390/ijms24086922

**Published:** 2023-04-07

**Authors:** Aleksandra Stupak, Tomasz Gęca, Anna Kwaśniewska, Radosław Mlak, Paweł Piwowarczyk, Robert Nawrot, Anna Goździcka-Józefiak, Wojciech Kwaśniewski

**Affiliations:** 1 Chair and Department of Obstetrics and Pathology of Pregnancy, Medical University of Lublin, 20-059 Lublin, Poland; 2 Body Composition Research Laboratory, Department of Preclinical Science, Medical University of Lublin, 20-059 Lublin, Poland; 3 2nd Department of Anesthesiology and Intensive Care Unit, Medical University of Lublin, 20-059 Lublin, Poland; 4 Department of Molecular Virology, Institute of Experimental Biology, Adam Mickiewicz University in Poznan, 61-712 Poznań, Poland; 5 Department of Gynecologic Oncology and Gynecology, Medical University of Lublin, 20-059 Lublin, Poland

**Keywords:** microbiome, bacteria, proteome, pregnancy, FGR, placenta

## Abstract

A comparative analysis of the placental microbiome in pregnancies with late fetal growth restriction (FGR) was performed with normal pregnancies to assess the impact of bacteria on placental development and function. The presence of microorganisms in the placenta, amniotic fluid, fetal membranes and umbilical cord blood throughout pregnancy disproves the theory of the “sterile uterus”. FGR occurs when the fetus is unable to follow a biophysically determined growth path. Bacterial infections have been linked to maternal overproduction of pro-inflammatory cytokines, as well as various short- and long-term problems. Proteomics and bioinformatics studies of placental biomass allowed the development of new diagnostic options. In this study, the microbiome of normal and FGR placentas was analyzed by LC-ESI-MS/MS mass spectrometry, and the bacteria present in both placentas were identified by analysis of a set of bacterial proteins. Thirty-six pregnant Caucasian women participated in the study, including 18 women with normal pregnancy and eutrophic fetuses (EFW > 10th percentile) and 18 women with late FGR diagnosed after 32 weeks of gestation. Based on the analysis of the proteinogram, 166 bacterial proteins were detected in the material taken from the placentas in the study group. Of these, 21 proteins had an exponentially modified protein abundance index (emPAI) value of 0 and were not included in further analysis. Of the remaining 145 proteins, 52 were also present in the material from the control group. The remaining 93 proteins were present only in the material collected from the study group. Based on the proteinogram analysis, 732 bacterial proteins were detected in the material taken from the control group. Of these, 104 proteins had an emPAI value of 0 and were not included in further analysis. Of the remaining 628 proteins, 52 were also present in the material from the study group. The remaining 576 proteins were present only in the material taken from the control group. In both groups, we considered the result of ns prot ≥ 60 as the cut-off value for the agreement of the detected protein with its theoretical counterpart. Our study found significantly higher emPAI values of proteins representative of the following bacteria: *Actinopolyspora erythraea*, *Listeria costaricensis*, *E. coli*, *Methylobacterium*, *Acidobacteria bacterium*, *Bacteroidetes bacterium*, *Paenisporsarcina* sp., *Thiodiazotropha endol oripes* and *Clostridiales bacterium*. On the other hand, in the control group statistically more frequently, based on proteomic data, the following were found: *Flavobacterial bacterium*, *Aureimonas* sp. and *Bacillus cereus*. Our study showed that placental dysbiosis may be an important factor in the etiology of FGR. The presence of numerous bacterial proteins present in the control material may indicate their protective role, while the presence of bacterial proteins detected only in the material taken from the placentas of the study group may indicate their potentially pathogenic nature. This phenomenon is probably important in the development of the immune system in early life, and the placental microbiota and its metabolites may have great potential in the screening, prevention, diagnosis and treatment of FGR.

## 1. Introduction

The human body is inhabited by numerous microorganisms that constitute a kind of microbiome. The importance of microorganisms inhabiting various parts of the human body is not fully understood. Little is known about their impact on human growth, development and health. There is particularly little data on the impact of bacteria and viruses on the development of the placenta in normal and complicated pregnancies, e.g., with fetal growth disorders.

Fetal growth retardation, observed in approximately 3–10% of pregnancies, is one of the problems of perinatology, whose etiology and pathogenesis are not fully understood [1,2]. FGR is most commonly defined as the estimated fetal weight below the 10th percentile for gestational age based on prenatal ultrasound assessment [3]. This condition is associated with a number of short-term and long-term complications that can seriously affect the quality of life [4].

About 40% of FGR cases are idiopathic with no identifiable cause. In the remaining 60% of cases, 1/3 of intrauterine growth retardation is caused by genetic abnormalities and 2/3 is induced by environmental factors [5]. Factors affecting the development of intrauterine growth retardation can be divided into four groups: maternal factors, placental factors, fetal factors and infectious factors [6,7,8].

So far, cases of FGR have been documented with bacterial infections such as mycoplasma, listeria and mycobacteria, tuberculosis, and infection with a virulent, pathogenic *E. coli* strain. There are also indications that the composition of the vaginal flora may increase the likelihood of FGR. Studies have shown that the simultaneous presence of *Bacteroides*, *Prevotella*, *Porphyromonas* spp., *M. hominis*, *U. urealyticum* and *T. vaginalis* doubles the likelihood of FGR [9]. The presence of bacterial and viral infections causes overproduction of pro-inflammatory cytokines in the mother’s body, such as interferon, tumor necrosis factor (TNF) or interleukins [10]. This causes widespread inflammation and necrosis, which in the case of the placenta can lead to abnormal distribution of nutrients and oxygen.

The state of the microenvironment of the maternal-fetal unit and its impact on the course of pregnancy, delivery and further health of the child and adult has been controversial for many years. They resulted from technical difficulties related to sampling and their analysis (biomass samples with a low content of microorganisms could be dominated by contamination during sampling or DNA isolation). On the other hand, in the studies carried out so far, we observe a very high methodological heterogeneity of the methods used to detect bacteria and viruses in placental tissues. This applies, for example, to the selection of a sequencing platform, DNA isolation kits and the selection of variable regions of the 16sRNA gene, which affects the ambiguity of the analyzed results. The introduction of new generation sequencing (NGS), mass spectrophotometry, proteomics and bioinformatics analysis of the obtained results enabled new diagnostic possibilities, primarily high sensitivity of biomass diagnostics of the tested material, e.g., bearing.

The presence of microorganisms in the placenta, amniotic fluid, fetal membranes and umbilical cord blood in studies using next-generation DNA sequencing technology undermines the sterility of the intrauterine environment during pregnancy and at the same time refutes the “sterile uterus” hypothesis, which was considered formulated in the early 20th century. The consequences of the presence of bacteria in the uterus are far-reaching in medicine and basic sciences and shed new light on the antibiotic treatment of pregnant women. It has been shown that the state of the biomass of the uterine environment later affects the development of atopy, asthma, allergies and obesity [11,12,13].

There are two working definitions of the “microbiome”. The first definition given by *Nature* defines the “microbiome” as “*all the genetic material it contains*” (*microbiota—the entire collection of microorganisms in a specific niche, such as the human gut*). This can also be called the metagenomic microbiome [14]. The second definition proposed by Whipps et al. defines “microbiome” as “*a distinctive microbial community occupying a fairly well-defined habitat that has distinct physicochemical properties*” [15]. So, the term doesn’t just refer to the microorganisms involved, it also covers their mechanisms of action. Both definitions are linked by emphasizing the functional capacity of the microbiome and the resulting activity [16].

The best-known microbiome is the microbiome of the digestive system, in which, apart from potentially pathogenic organisms, bacteria beneficial to metabolism and human health have been identified [17,18,19]. The microbiome is also called our “second genome” because it is the genome of the microorganisms that inhabit our bodies.

Since 2007 the human microbiome has been studied by the *Human Microbiome Project* (*HMP*) [20]. These studies made it possible to characterize the normal flora of the female genital organ [21]. The results of HMP and Sirota show a low diversity of genital micropopulations with a predominance of *Lactobacillus bacteria cillus* with a slight predominance of *Provotella*, *Grdnerella* and *Atopobium* [20,21,22]. These species form a “physiological flora” and by their presence prepare the endometrium from embryo implantation to the ability to carry a fetus to term [23].

It is now believed that the baby’s microbiome is influenced by both the mother’s microbiome and the immediate external environment. Given the important role of the microbiome, it is crucial to know and understand the mechanisms of colonization of the newborn. It has been suggested that the first colonization of the fetus occurs via the placental microbiome, but there is no clear evidence for this [24,25]. Hemochorial placentas found in humans are characterized by high permeability to lipophilic substances, contain a protein-mediated transport system for glucose and amino acids, exhibit exocytosis and endocytosis, and are permeable to hydrophilic substances through pores that can be used for bacterial migration [24,26].

## 2. Results

The aim of the presented study was to analyze and compare the microbiome of normal and FGR placentas using proteomic methods. In our studies, bacteria present in normal and FGR placentas were identified based on the analysis of a set of bacterial proteins (bacterial proteome) present in the examined clinical material.

### 2.1. Characteristics of the Control and Study Group

The results of the study were obtained from 18 placentas taken from women with fetal growth disorders and from 18 control placentas. The clinical characteristics along with anthropometric measurements of mothers and their newborns are presented in our previous study, as in Table 1 [27].

There were no statistically significant differences between the research groups in terms of age, height, fertility, BMI prior to pregnancy and body weight before pregnancy and at delivery. The only statistically significant difference between the two groups was that the control group gained significantly more weight during pregnancy than the experimental group (*p* = 0.032). In the study group, the mean pulsation index (PI) in the gestational uterine arteries was statistically substantially greater than in the control group (*p* = 0.025), as was the PI in the arterial umbilical cord of fetuses with FGR compared to eutrophic fetuses (*p* = 0.0001). The CPR was substantially greater in the control group than in the study group (*p* = 0.0005). Women in the experimental group gave birth much earlier than those in the control group (*p* = 0.001). Compared to neonates from the control group, infants with FGR had a lower birth weight (*p* = 0.0001), shorter body length (*p* = 0.001), and poorer Apgar score in the first minute of life (*p* = 0.002).

### 2.2. Analysis of the Bacterial Proteome in the Study and Control Groups

**Figure 1:**
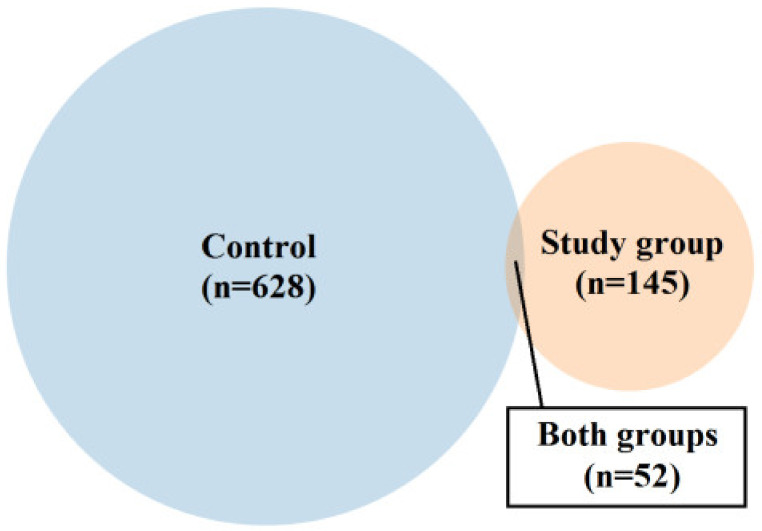
Venn diagram showing the proportion between the number of proteins (for which emPAI values > 0) identified only in the material representing the control or study group and present simultaneously in both types of the samples.

**Figure 2:**
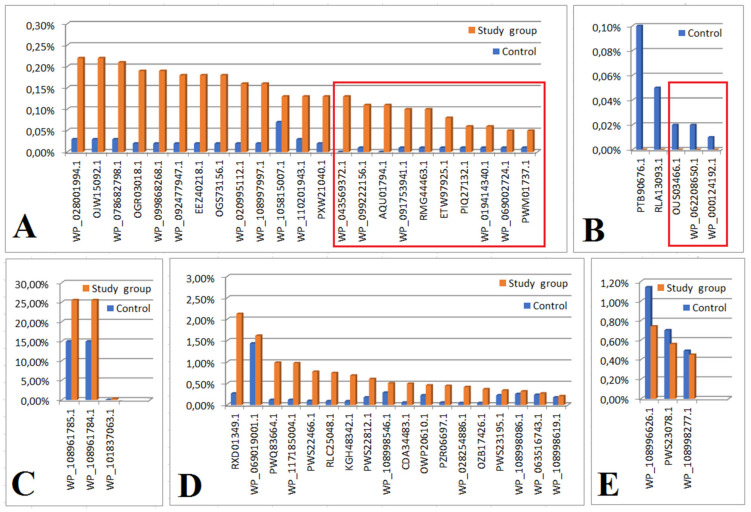
Bar graphs showing the comparison of proteins present in both the test group and the control group. Statistically significant results that remained significant after applying the Bonferroni correction are marked with a red square. Graph showing protein content comparisons for which emPAI values were: significantly higher in the study group compared to control (**A**); significantly higher in control compared to the study group (**B**); not significantly higher in the study group compared to the control (the results, however, trend toward significance) (**C**); not significantly higher in the study group compared to control (**D**); not significantly higher in control compared to study group (**E**). Expressions from Figure 2 are explained in Table 2.

**Figure 3:**
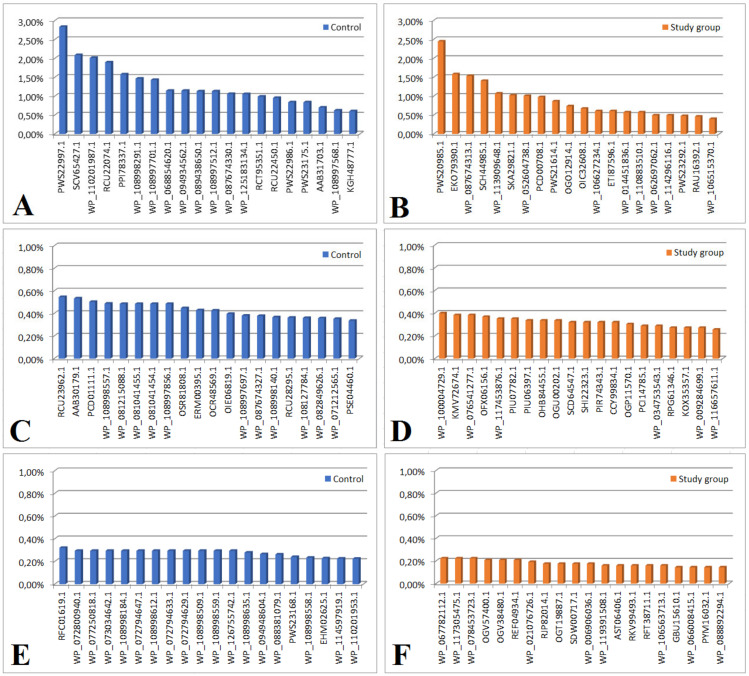
Bar graphs showing the content of proteins present only in the material from the study group or control. Graph showing the content of proteins (in order of decreasing emPAI value) found only in the material from the control group: proteins 1–20 (**A**); proteins 21–40 (**C**); proteins 41–60 (**E**) or tested: proteins 1–20 (**B**); proteins 21–40 (**D**); proteins 41–60 (**F**). Expressions from Figure 3 are explained in Table 3.

In both groups, we considered an ns prot score ≥ 60 as a cut-off value for the detected protein’s agreement with its theoretical equivalent. Based on the analysis of the proteinogram in the material collected from people from the study group (n = 18), 166 bacterial proteins were detected. Of these, 21 proteins had an emPAI value of 0 and were not included in further analysis. Out of the remaining 145 proteins, 52 were also present in the material from the control group (the differences in their content in individual materials are presented in Figure 1 and Figure 2, and Table 2 and Table 3). The remaining 93 proteins were present only in the material collected from the study group (their content in the material is shown in Figure 3B,D,F and Supplementary Figure S1B,D).

Based on the proteinogram analysis, 732 bacterial proteins were detected in the material collected from the control group (n = 18). Of these, 104 proteins had an emPAI value of 0 and were not included in the further analysis. Of the remaining 628 proteins, 52 were also present in the material from the study group. The remaining 576 proteins were present only in the material collected from the control group (their content in the material is shown in Figure 3A,C,E and Supplementary Figures S1A,C,E and S2–S5).

Based on proteomic data, the bacteria identified in both groups are presented in Table 4 and Table 5.

Based on proteomic data, the bacteria identified in both groups, but significantly higher in the study group, are: *Actinopolyspora erythraea*, *Listeria costaricensis*, *E. coli*, *Methylobacterium*, *Acidobacteria bacterium*, *Bacteroidetes bacterium*, *Paenisporsarcina* sp., *Thiodiazotropha endolloripes* and *Clostridiales bacterium*. The tendency towards statistical significance also concerned *Klebsiella* sp.

In addition, bacteria that may be pathological were identified in the study group (although data insignificantly higher): *Splinomonas* sp., *Pseudoalteromonas*, *Stenoprophomonas maltophila*, *Pseudomonas cichorii*, *Enterococcus faecium*, *Deltaproteobacteria bacterium*, *Acinetobacter baumannii*, *Firmicutes bacterium*, *Microbacterium*, *Archangium gephora*, *Veillonella magna*, *Hyphomonas* sp., *Enterococcus faecium* and *Lactobacillus harbinensis*.

In turn, in the control group, statistically more often, based on proteomic data, the following were found: *Flavobacterial bacterium*, *Aureimonas* sp., *Bacillus cereus* and *Klebsiella* were on the verge of statistical significance, as well as *Pneumoniae*, *Enterococcus faecium* and *E. coli*.

## 3. Discussion

Advances in molecular methodology reveal the details of the human-microbial relationship, allowing for increased identification of microbiota composition and function. Recently, the maternal microbiome has been shown to prepare the newborn for host–microbial symbiosis, driving postnatal innate immune development [28]. However, the viability of placental bacteria cannot be determined due to discrepancies with the culture results [24]. On the other hand, some of these microorganisms may not be easily cultured, but they can be detected by DNA analysis.

The results of a review evaluating the microbiological composition of the placenta in a healthy pregnant woman and the potential relationship between the placental microbiome and the oral microbiome have shown the existence of a low biomass placental microflora in pregnant women with a normal course of pregnancy [29].

In turn, animal studies have shown that despite differences in gut physiology and morphology, both humans and cattle require a functional microbiome early in life (pre-implantation and organogenesis) and throughout pregnancy [25]. Studies indicate that both species acquire intestinal microbes before birth, possibly from the mother, which would indicate the existence of similar mechanisms and timing of fetal intestinal colonization.

Other studies have shown that gut microbiota dysbiosis is an important etiology of pre-eclampsia (PE) [30,31]. The intestinal microbiota and its active metabolites have great potential in the treatment and diagnosis of PE. The results of the cited work enrich the theory of the enteroplacental axis and contribute to the development of microecological products for preeclampsia. PE and FGR are placental-mediated disorders, and metabolomic studies of maternal-fetal pairings may aid in understanding their pathogenesis. Microbiome profiles from 37 overweight and obese pregnant women enrolled in the SPRING cohort were examined by 16SrRNA sequencing [32,33]. Consistent with our findings, four main bacterial phyla (*Firmicutes*, *Bacteroidetes*, *Actinobacteria* and *Proteobacteria*) were identified in all microbiomes. The possible origin of the placental microbiome was both the maternal oral and gut.

FGR is a complex obstetric complication with various causes and a wide spectrum of complications, especially for the fetus, as it is associated with an increased risk of perinatal mortality and morbidity. As highlighted above, the pathogenesis of FGR is unclear, which limits its effective treatment. It has been found that the dysbiosis of the intestinal microflora plays an important role in the pathogenesis of various diseases. However, its role in the development of FGR remains unclear and requires clarification.

In our study, significantly higher in the study group were bacteria: *Actinopolyspora erythraea*, *Listeria costaricensis*, *E. coli*, Methylobacterium, *Acidobacteria bacterium*, *Bacteroidetes bacterium*, *Paenisporsarcina* sp., *Thiodiazotropha endoloripes* and *Clostridiales bacterium*. On the other hand, in the control group, statistically more frequently, based on proteomic data, the following were found: *Flavobacterial bacterium*, *Aureimonas* sp. and *Bacillus cereus*.

Correlations between, e.g., *Helicobacteria pylori*, and the development of FGR in a group of 600 women were demonstrated by den Hollender et al. [34]. In turn, the important factor, which is the intestinal microbiome of infants, is indicated by the results of research by Groer et al. and Yang et al. [35]. Yang’s research has shown correlations between an infant’s physical development and fecal cysteine concentrations [36]. It also turned out that *Oscillospira* and *Coprococcus* are involved in the synthesis of butyrate, which is a source of energy for intestinal epithelial cells. Consistent with our results, a study by Tu et al., evaluating the feces of infants with FGR, showed significant differences in the growth of *Bacteroides*, *Faecalibacterium* and *Lachnospira* in patients with growth restriction [37].

In a pilot study by Hu et al., the relationship of FGR with the reproductive microbiome has been studied [38]. The reproductive microbiome was studied by 16sRNA sequencing (20-IUGR, 20-controls). Microbiological screening of the placenta showed a diverse flora as in our results, mainly *Proteobacteria*, *Fusobacteria*, *Firmicutes* and *Bacteroidetes*. The study group with FGR was characterized by a higher incidence of β-hemolytic bacteria *Neisseriaceae* and an increase in the number of anaerobic bacteria *Desulfovibrio* reflective of placental hypoxia. Further analysis of the reproductive microbiome of the FGR samples revealed lower levels of H2O2-producing *Bifidobacterium* and *Lactobacillus* that go from respiration to fermentation, a less energetic metabolic process as oxygen levels drop. Source tracing analysis showed that placental microbial content was predominantly from an oral source, compared to an intestinal or vaginal source. The cited results suggest that reproductive microbiome profiles may be potential biomarkers for fetal health during pregnancy in the future, while *Neisseriaceae* may represent promising therapeutic targets for the treatment of IUGR.

The *Actinopolyspora erythraea* protein identified in our FGR placentas catalyzes the circularization of gamma-N-acetyl-alpha, gamma—diaminobutyric acid (ADABA) to ectoine (1,4,5,6-tetrahydro-2-methyl-4-pyrimidine carboxylic acid), an effective osmoprotectant [39]. This prokaryote occupies an “extreme or inhabitable environment” [40]. These bacteria (extremophiles) have evolved to harsh pH, temperature, salinity and pressure by biosynthesizing unique compounds, such as new enzymes. *Acidobacteria* appear to be able to resist numerous pollutants, such as PCBs and petroleum compounds, linear alkylbenzene sulfate, p-nitrophenol, and heavy metals, under low pH circumstances [41]. A high number of acidobacterial genes code for transporters belonging to the drug/metabolite transporter superfamily. Unfortunately, no data supporting real actions linked to pollution degradation have been documented. The role of these bacteria in the pathophysiology of FGR is unknown.

*Methylobacterium* was also identified in FGR placentas; it is an emerging opportunistic premise plumbing pathogen (OPPsP) [42]. It possesses chlorine resistance, biofilm development, desiccation tolerance, and resilience to temperatures above 50 degrees Celsius. *Methylobacterium extorquens*, like other OPPPs, was isolated from amoebae in drinking water systems, making it an amoeba-resistant bacteria.

The sophisticated methods used in our research are based on the identification of proteins using LC-ESI-MS/MS by pooling material from 18 FGR and 18 control placentas. To distinguish between placental samples and contamination introduced during DNA extraction, purification and amplification, unsupervised ordination methods showed a separate clustering between pooled negative control and placental samples like in studies performed by others [32]. These methods do not distinguish between live, dead or ruptured bacterial fragments. Differences in relation to the data of other researchers may be statistically significant because the statistical analysis does not concern individual cases but material from the studied population, which seems to be more convincing in terms of the population.

The clinical implication of our research could be a careful consideration in the rational prescription and use of antibiotics to avoid infections while at the same time protecting the fetus from the adverse effects of pharmacotherapy.

A limitation of our study would be that we did not perform any bacterial culture of the tested bearings due to the presence of potentially viable bacteria. The material for the study was collected during cesarean section in sterile conditions. However, other researchers have confirmed that this method is devoid of the possibility of contamination [43]. Moreover, none of the taxa of bacteria mentioned in other studies were found to have different abundance between vaginally delivered and cesarean placentas [32].

Another limitation of our work is that we did not collect reference material for microbiome analysis from other parts of the body of pregnant women, such as saliva, vaginal secretions or feces. Our main goal was to determine the occurrence of individual bacteria traces, not their origin. However, it is known that the placental core microbiome shares phylotypes with the maternal oral and gut microbiome [32].

## 4. Materials and Methods

Patients hospitalized at the Department of Obstetrics and Pathology of Pregnancy at the Medical University of Lublin between 2019 and 2021 and between 32 and 36 weeks’ gestation with a singleton pregnancy and late-onset FGR were selected for the research. Multiple pregnancies, the presence of any antenatal infections, a positive TORCH test result, treatment with antibiotics during pregnancy, any form of hypertension in pregnancy, pre-pregnancy and gestational diabetes, nephropathy, thyroid dysfunction and any other general diseases before pregnancy, the use of any drugs or stimulants, cigarette smoking, and fetuses with birth defects and chromosomal abnormalities were excluded from the study.

Thirty-six pregnant Caucasian women participated in the study, comprising 18 women with physiological pregnancy and eutrophic fetus (EFW > 10th percentile) (control group) and 18 women with late FGR identified after 32 weeks of pregnancy, according to Delphi consensus (study group) [3]. Placenta samples were successfully obtained from all eligible participants. Hadlock et al. devised a regression equation using the biparietal diameter, the length of the femur, and the head and belly circumferences to estimate the fetal weight during an ultrasound examination [44]. During one week before the birth, Doppler measurements of the umbilical artery free loop were taken using a Voluson E9 with RA4B 3D 4–8 MHz curvilinear probe (GE Healthcare, Hatfield, UK). Then, the pulsatility index (PI), resistance index (RI) and cerebroplacental ratio (CPR) were computed. PI = (S − D)/A and RI = (S − D)/S, where S represents the systolic peak, D represents the end-diastolic flow, and A represents the temporal average frequency. In contrast, the CPR is the ratio between the PI of the middle cerebral artery (MCA) and the umbilical artery (UA) (PI MCA/PI UA) and reflects the distribution of cardiac output in favor of cerebral blood flow. It is one of the criteria with the highest predictive accuracy for perinatal outcomes [45]. In response to intrauterine hypoxia, fetal blood flow is redistributed to the brain, and the value of CPR reduces by 1. In cases of late-onset FGR, hypoxia tolerance is lower than in cases of early-onset FGR [46].

Using standardized medical records and patient interviews, smoking, age, weight, and body mass index (BMI) at the beginning of the first trimester, pregnancy weight increase, and TORCH were determined for the mothers. The BMI was computed by dividing body weight (kg) by height (m^2^). Moreover, data including information on infants: gestational age at delivery, gender and birth weight of the newborn, placental weight, body length, head circumference, and neonatal problems. The gestational age was calculated using the latest menstrual period and the first-trimester ultrasonography (based on crown–rump length (CRL)). Immediately after birth, placenta weight and the neonate birth weight, body length, and head circumference were measured using the proper measuring instruments.

Material for proteomic investigation consisted of pieces of normal placentas serving as controls and fragments of placentas obtained from mothers with FGR. Following the process, trained employees collected all samples. During the cesarean section, soon after childbirth, the placenta was put in sterile containers containing ice under aseptic circumstances. Those responsible for collecting specimens wore a sterile protective apron, face masks and sterile gloves to guarantee sterility throughout the sampling procedure. The placentae were collected and weighed. Four placenta samples measuring 1.0 × 1.0 × 1.5 cm were taken from each placenta (overall number of samples: 144), around 3 to 4 cm from the umbilical cord attachment point, from four separate quadrants of the placenta. To reduce the possibility of infection after cesarean delivery, only portions from the inner placenta were obtained for evaluation (risk of contamination). Each placenta sample was put in a sterile, labeled cryovial, frozen in liquid nitrogen, and kept at −80 degrees Celsius for future study.

### 4.1. Identification of Proteins Using LC-ESI-MS/MS

Liquid chromatography–mass spectrometry (LC-MS/MS) is an exceedingly sensitive and specific analytical technique that can precisely determine the identities and concentration of compounds within samples [47]. Because it identifies the proteins that are present in a sample and quantifies the abundance levels of the discovered proteins, it is utilized in proteomic research.

#### 4.1.1. Protein Extraction

One hundred milligram sections were taken from the collected clinical material. Eighteen sections from normal placentas (control group) and eighteen FGR placentas (study group) were pooled and proteins were isolated. Clinical material in both samples was homogenized in liquid nitrogen (by mechanical homogenization in liquid nitrogen) and then suspended in a solution of 0.2 M (NH_4_)_2_SO_4_ and left on ice for 30 min. After this time, the samples were centrifuged (10,000× *g*) for 39 min to remove cell debris. Proteins were isolated from the supernatant according to the procedure described by Diffley [48]. The quality of the preparations was assessed using 1-D gel electrophoresis separation [49].

#### 4.1.2. Mass Spectrometry Examination

In the Laboratory of Mass Spectrometry, Institute of Biochemistry and Biophysics, Polish Academy of Sciences, Warsaw, Poland, proteins were examined by liquid chromatography linked to a mass spectrometer. Using two technical replicates, tryptic peptide mixtures were evaluated by LC-ESI-MS/MS employing nanoflow HPLC and an LTQ-Orbitrap XL (Thermo Fisher Scientific, Waltham, MA, USA) as the mass analyzer. Trypsin was used to break down the proteins. The synthesized peptides were concentrated, desalted on an RP-C18 precolumn (LC Packings, Coventry, UK), and then separated by UltiMate nano-HPLC (LC Packings, San Diego, CA, USA) with a linear acetonitrile gradient (from 10 to 30%) over 50 min. The column was linked directly to a nanospray ion source working in a data-dependent MS to MS/MS mode transition. Proteins were identified by tandem mass spectrometry (MS/MS) by acquiring fragmentation spectra of multiple-charged peptides in a manner dependent on information.

#### 4.1.3. Identification Method for Proteins

MASCOT 2.4.1 (Matrix Science, London, UK) was used to search the Uniprot 2019_02 (561356 sequences; 201858328 residues) database with bacterial sequences and a filter to examine the spectrum data. These were the search criteria for mascots: With variable carbamidomethyl (C) and oxidation (M) modifications, peptide mass tolerance of 50 ppm and fragment mass tolerance of 0.8 Da. Acceptable protein identification required the identification of at least two peptide fragments per protein.

#### 4.1.4. Quantitative Evaluation

The Exponentially Modified Protein Abundance Index (emPAI) was utilized to perform a non-label quantitative comparison of proteins between studied samples [50]. The protein abundance index (PAI) is the quantity of peptides per protein normalized by the theoretical number of peptides. The exponential version of PAI minus one (emPAI = 10PAI 1) is used to calculate protein abundance from nano-LC–MS/MS investigations. The value of emPAI is proportional to the abundance of proteins in a protein mixture. In Excel files, the resulting protein and peptide lists were preserved.

### 4.2. Statistical Analysis

The statistical analysis of the data collected in the spreadsheet was carried out using the Statistica program (v. 13 PL, TIBCO Software Inc. Palo Alto, CA, USA). The content (expressed by percentages calculated based on the emPAI value) of individual bacterial proteins in the test material (derived from the study group or control) was compared using the *Chi*-squared test with Yates’ correction. All reported *p*-values are two-sided (or two-tailed). Since the analysis was based on multiple comparisons, Bonferroni correction was applied. Thus, only results with p < 0.001 were considered statistically significant.

### 4.3. Ethics

Written informed consent was obtained from all subjects included, and the study was performed in accordance with the principles of the Helsinki Declaration. The research was issued by the Bioethics Committee at the Medical University of Lublin (Approval No. KE-0254/87/2020). Derived data supporting the findings of this study are available from the corresponding author on request.

## 5. Conclusions

To sum up, the presence of numerous bacterial proteins present in the control material, which are absent in the material taken from the study group, may indicate their protective role. Similarly, the presence of bacterial proteins found only in the material obtained from people from the study group may indicate their potentially pathogenic nature.

However, we must remember that the proteome does not necessarily correspond to the content of the bacteria themselves, due to the variable expression of many proteins depending on their type and needs.

## Figures and Tables

**Table 1:** Clinical characteristics and anthropometric measurements of mothers and newborns.

	Control Group N = 18 Median (Range)	Study Group (FGR) N = 18	*p*-Value
Baseline characteristics			
age (years)	30.2 ± 6.5	28.2 ± 5.6	0.466
height (m)	1.7 ± 0.06	1.67 ± 0.08	0.373
Actual weight (kg)	80.3 ± 11.3	79.5 ± 7	0.854
Weight before pregnancy (kg)	63.6 ± 11.3	66.7 ± 6.3	0.458
BMI before pregnancy (kg/m^2^)	22 ± 3.5	23.9 ± 2.1	0.154
weight gain (kg)	14 (12–28)	12.5 (11–15)	0.032 *
weight of the placenta (g)	515 ± 46	328 ± 53	<0.001 *
Parity	2 (1–4)	1 (1–3)	0.504
Gestation	2 (1–4)	1.5 (1–4)	0.699
Perinatal outcomes			
Gestational age at the delivery (weeks)	39 (38–41)	37 (35/4–40)	0.002 *
Fetal weight at birth (g)	3540 (2910–3890)	2300 (1385–2570)	<0.001 *
neonatal length (cm)	54 (47–57)	48 (35–51)	0.001 *
APGAR 1 min (points)	9 (min. 8–max. 10)	8 (min. 6–max. 9)	0.002 *
APGAR 5 min (points)	10 (min. 9–max. 10)	10 (min. 6–max. 10)	0.597
Feto-placental Doppler before delivery			
AU PI	0.77 (0.72–0.91)	1.11 (0.98–1.9)	<0.001 *
MCA PI	1.44 ± 0.21	1.31 ± 0.22	0.191
UTA PI	0.79 ± 0.05	0.93 ± 0.17	0.025 *
CPR	1.703 (1.48–2.444)	0.995 (0.737–1.687)	<0.001 *

Values are shown as median (interquartile range) or mean ± standard deviation; statistical analysis was performed using a Mann–Whitney U test. Body mass index (BMI), pulsatility index (PI), umbilical artery (UA), middle cerebral artery (MCA), uterine artery (Ut A), cerebro-placental ratio (CPR), *—statistically significant results.

**Table 2:** Legend to Figure 2.

Accession number	Protein (*Bacteria*)
WP_043569372.1	catalase (*Actinopolyspora erythraea*)
WP_099222156.1	HTH domain-containing protein (*Listeria costaricensis*)
AQU01794.1	glucose-6-phosphate isomerase (*Escherichia coli*)
WP_091753941.1	AAA family ATPase (*Methylobacterium* sp. ap11)
RMG44463.1	hypothetical protein D6718_10020 (*Acidobacteria bacterium*)
ETW97925.1	hypothetical protein ETSY1_20835, partial (*Candidatus entotheonella* factor)
PIQ27132.1	DNA polymerase I (*Bacteroidetes bacterium* CG18_big_fil_WC_8_21_14_2_50_41_14)
WP_019414340.1	hypothetical protein (*Paenisporosarcina* sp. TG20)
WP_069002724.1	DUF1631 family protein (*Candidatus Thiodiazotropha endoloripes*)
PWM01737.1	hypothetical protein DBY05_04045 (*Clostridiales bacterium*)
WP_28001994.1	shikimate dehydrogenase (*Shinorhizobium arboris*)
GCI 15092.1	phytoene synthase (*Mucilagini bacteria* sp.)
WP_078682797.1	dTDP-4dehydrorramnose reductase (*Lentisphaerae bacteria* GWF-2)
OGR030118.1	hypothetical protein (*Pararhizbium haloflavum*)
WP_92477947.1	A/G-specific adenine glycolase (*Clostridium polysaccharoliticum*)
EEZ40218.1	derythrose-4-phosphate dehygrogenase (*Photobacterium damselae subs daselae*)
OGS73156.1	flagellar biosynthesis protein F1hB (*Gallionellales bacterium* GWAZ)
WP_020995112.1	hypothetical protein (*Oxalobacter formigenes*)
WP_108997997.1	4-hydroxybenzoate-3-monooxygenase (*Salinibacterium* sp.)
WP_105815007.1	hypothetical protein (*Mycobacterium tuberculosis*)
WP_110201943	protein disulfide isomerase (*Kangiella* sp.)
PXW21040	activator of mannose operon (transcriptional terminatol) (*Pantoea* sp.)
Figure 2B	
OUS03466.1	hypothetical protein A9Q86_00710 (*Flavobacteriales bacterium* 33_180_T64)
WP_062208650.1	tryptophan--tRNA ligase (*Aureimonas* sp. AU12)
WP_000124192.1	S8 family peptidase (*Bacillus cereus*)
PTB9-676.1	hypothetical protein (*Marvigra lubricoides*)
RLA13093.1	uracil DNA glucose (*Gammaproteobacteria bacterium*)
Figure 2C	
WP_108961785	hypothetical protein (*E. coli*)
WP_108961784.1	hypothetical protein (*E. coli*)
WP_101837063.1	hypothetical protein (*Klebsiella* sp.)
Figure 2D	
RXD 01349.1	ubiquitin (*Splinomonassp*)
WP_069019000.1	actin cytoplasmic (*Pseudoalteramonas* sp.)
PWQ83644.1	30 Sribosomal oritein S15, partial (*Stenoprophomonas maltophilla*)
WP_1171850004.1	hypothtical protein (*Pseudomonas chorii*)
PWS22466.1	hypothtical protein PKP2260 (*Enterococcus faecium*)
RLC25048.1	hypothetical protein DRX56 (*Dettaproteobacteria bacterium*)
KGH48342.1	hypothetical protein GS19 (*Acinetobacter baumans*)
PWS22812.1	hypothetical protein PKP2260 (*Enterococcus faecium*)
CDA34483.1	Predicted DNA-binding protein withPD1 like DNA binding motif (*Firmicutes bacterium* CAG-536)
OWP2061.01	hypothetical protein CBF 90 (*Microbacterium sp.*)
PZR06697.1	hypothetical protein DI536 (*Archangium gephora*)
WP_028254886.1	BREX-3-SYSTEMP loop-containing protein BrxF (*Vellonella magna*)
OZB17426.1	ribosomal recycling factor (*Hyphomonas* sp.)
PWS233195.1	hypothetical protein DKP78 (*Enterococcus faecium*)
WP_108998086.1	NADP+ isocitrinate dehydrogenase (*Escherichia coli*)
WP_063516743.1	molecular chaperone Htp G (*Lactobacillus harninensis*)
WP_108998619.1	lactate dehydrogenase (*E. coli*)
Figure 2E	
WP_108996626.1	hypothetical protein (*Klebsiella pneumoniae*)
PWS23078.1	hypothetical protein (*Enterococcus faecium*)
WP_108998277.1	malate dehydrogenase (*E. coli*)

**Table 3:** Legend to Figure 3.

Protein content > 1%	
PWS22997.1	hypothetical protein DKP78_15465, partial (*Enterococcus faecium*)
RCU22074.1	hypothetical protein DVA69_20680, partial (*Acinetobacter baumannii*)
WP_087674330.1	MULTISPECIES: peptidylprolyl isomerase (*Gammaproteobacteria*)
SCV65427.1	Core histone H2A/H2B/H3/H4 (*Anaplasma phagocytophilum*)
WP_110201987.1	actin, cytoplasmic 2 (*Kangiella spongicola*)
PPI78337.1	actin, cytoplasmic 2, partial (*Marinobacter flavimaris*)
WP_108998291.1	50S ribosomal protein P1 (*E. coli*)
WP_108997701.1	F0F1 ATP synthase subunit beta (*E. coli*)
WP_068854620.1	hypothetical protein (*Klebsiella pneumoniae*)
WP_094934562.1	hypothetical protein (*Klebsiella pneumoniae*)
WP_089438650.1	actin, cytoplasmic 2 (*E. coli*)
WP_108997512.1	actin, cytoplasmic 2 (*E. coli*)
WP_125183134.1	hypothetical protein, partial (*Enterobacter hormaechei*)
Figure 3B Protein content > 1%	
PWS20985.1	hypothetical protein DKP78_25965, partial (*Enterococcus faecium*)
WP_087674313.1	hypothetical protein (*Pseudomonas syringae*)
SCH44985.1	Uncharacterized protein (uncultured *Clostridium* sp.)
PCD00708.1	hypothetical protein CO192_04000, partial (*Pseudomonas pelagia*)
ECO79390.1	hypothetical protein LEP1GSC068_2346 (*Leptospira sp. Fiocruz* LV3954)
WP_113909648.1	hypothetical protein (*Arcobacter* sp. FW59)
SKA29821.1	CheW-like domain-containing protein (*Oceanospirillum multiglobuliferum*)
WP_052604738.1	hypothetical protein (*Acidithrix ferrooxidans*)
Figure 3C	
RCU23962.1	Glu/Leu/Phe/Val dehydrogenase, partial (*Acinetobacter baumannii*)
AAB30179.1	p105 = epidermal keratin type 1 intermediate filament protein homolog {29 kda fragment} (*Mycoplasma*, Peptide Partial, 24 aa)
PCD01111.1	actin, cytoplasmic 2, partial (*Pseudomonas pelagia*)
WP_108998557.1	pyruvate kinase, partial (*E. coli*)
WP_081215088.1	hypothetical protein (*Lactococcus lactis*)
WP_081041455.1	hypothetical protein (*Lactococcus lactis*)
WP_081041454.1	hypothetical protein (*Lactococcus lactis)*
WP_1089977856.1	malate dehydrogenase (*E. coli*)
OSR81808.1	hypothetical protein BV331_05659 (*Pseudomonas syringae pv. actinidiae*)
ERM00395.1	hypothetical protein Q644_05090 (*Ochrobactrum intermedium* 229E)
OCR48569.1	hypothetical protein RJ97_26685, partial (*Klebsiella pneumoniae*)
OIE06819.1	hypothetical protein A7L78_18910 (*Acinetobacter baumannii*)
WP_108997697.1	fructose-bisphosphate aldolase class I, partial (*Escherichia coli*)
WP_087674327.1	50S ribosomal protein L10, partial (*Pseudomonas syringae*)
WP_108998140.1	nucleoside-diphosphate kinase, partial (*Escherichia coli*)
RCU28295.1	hypothetical protein DVA69_17570, partial (*Acinetobacter baumannlii*)
WP_108127784.1	tropomyosin (*Saccharospirillum mangrove*)
WP_082849626.1	molecular chaperone DnaK (*Lactobacillus harbinensis*)
WP_071212565.1	30S ribosomal protein S11, partial (*Acinetobacter baumannii*)
PSE04460.1	hypothetical protein C7G98_18875, partial (*Acinetobacter baumannii*)
Figure 3D engraving	
WP_10004729.1	integrase, partial (*E. coli*)
KMV72674.1	hypothetical protein AI28_14165 (bacteria symbiont BFo1 of *Frankliniella occidentalis*)
WP_076541277.1	DUF3833 domain-containing protein (*Shewanella sp*. UCD-KL21)
OFX06156.1	ATP:cob (I) alamine adenosyltransferase (*Alphaproteobacteria bacterium* RIFCSPHIGHO2_12_FULL_63_12)
WP_117453876.1	MULTISPECIES: hypothetical protein (*Absiella*)
PIU07782.1	hydrolase (*Methylobacterium sp*. CG09_land_8_20_14_0_10_71_15)
PIU06397.1	hypothetical protein COT56_11130 (*Methylobacterium sp*. CG09_land_8_20_14_0_10_71_15)
OHB84455.1	hypothetical protein A3J73_04470 (*Planctomycetes bacterium* RIFCSPHIGHO2_02_FULL_38_41)
OGU00202.1	thioredoxin peroxidase (*Geobacteraceae bacterium* GWC2_48_7)
SCD64547.1	transcriptional regulator, TetR family (*Streptomyces* sp. di50b)
SHI22323.1	NlpC/P60 family protein (*Leeuwenhoekiella palythoae*)
PIR74343.1	hypothetical protein COU35_02740 (*Candidatus Magasanikbacteria bacterium* CG10_big_fil_rev_8_21_14_0_10_47_10)
CCY99834.1	putative uncharacterized protein (*Clostridium sp*. CAG:793)
OGP11570.1	metal-dependent hydrolase (*Deltaproteobacteria bacterium* GWA2_43_19)
PCI14785.1	cell division ATP-binding protein FtsE (*Thiotrichales bacterium*)
WP_034753543.1	phosphoglycolate phosphatase (*Janthinobacterium liquid*)
RPG61346.1	lipoyl (octoyl) transferase LipB (*Flavobacteriaceae bacterium* TMED206)
KOX35357.1	HAD family hydrolase (*Saccharothrix* sp. NRRL B-16348)
WP_009284699.1	3-hydroxyacyl-CoA dehydrogenase (*Fibrisoma limit*)
WP_116657611.1	hydroxyacylglutathione hydrolase (*Pseudomonas* sp. NDM)
Figure 3E	
RFC01619.1	hypothetical protein DDJ49_30220, partial (*Klebsiella pneumoniae*)
WP_07280094.1	hypothetical protein (*E. coli*)
WP_077250818.1	hypothetical protein (*E. coli*)
WP_073034642.1	hypothetical protein (*E. coli*)
WP_108998184.1	hypothetical protein (*E. coli*)
WP_072794647.1	hypothetical protein (*E. coli)*
WP_108998612.1	hypothetical protein (*E. coli*)
WP_072794633.1	hypothetical protein (*E. coli*)
WP_072794629.1	hypothetical protein (*E. coli*)
WP_108998509.1	hypothetical protein (*E. coli*)
WP_108998559.1	hypothetical protein (*E. coli*)
WP_126755742.1	hypothetical protein (*E. coli*)
WP_108998635.1	60S ribosomal protein L22 (*E. coli*)
WP_094948604.1	MULTISPECIES: translation elongation factor EF-1 subunit alpha (*Enterobacteriaceae*)
WP_888381079.1	hypothetical protein (*Microbacterium sp*. AISO3)
PWS23168.1	hypothetical protein DKP78_14555, partial (*Enterococcus faecium*)
WP_108998558.1	30S ribosomal protein S19e (*E. coli*)
EHM02625.1	hypothetical protein HMPREF9946_00894 (*Acetobacteraceae bacterium* AT-5844)
WP_114597919.1	tubulin beta chain (*Microbacterium arborescens*)
WP_110201953.1	hypothetical protein (*Kangiella spongicola*)
Figure 3F	
WP_067782112.1	hypothetical protein (*Actinomyces vulturis*)
WP_117305475.1	MinD/ParA family protein (*Bacillus sp*. V59.32a)
WP_078453723.1	molecular chaperone DnaK, partial (*Solemya velum gill symbiont*)
OGV57400.1	hypothetical protein A2X49_01235 (*Lentisphaerae bacteria* GWF2_52_8)
OGV38480.1	transcriptional regulator (*Lentisphaerae bacteria* GWF2_49_21)
REF04934.1	LacI family transcriptional regulator (*Microbacterium chocolate*)
WP_021076726.1	magnesium chelate ATPase subunit I (*Bradyrhizobium sp.* MOS004)
RJP82014.1	MCE family protein (*Desulfobacteraceae bacterium*)
OGT19887.1	histidinol -phosphate transaminase (*Gammaproteobacteria bacterium* RBG_16_57_12)
SDW00717.1	hypothetical protein SAMN04487912_10192 (*Arthrobacter sp*. cf158)
WP_0069060036.1	phage major capsid protein (*Shuttleworthia satelles*)
WP_1193915508.1	pyrophosphate--fructose-6-phosphate 1-phosphotransferase (*Phyllobacteriaceae bacterium* SYSU D60012)
AST06406.1	MFS transporter (*Anoxybacillus flavithermus*)
RKV99493.1	type II secretion system F family protein (*Candidatus Saccharimonas sp.*)
RTF38711.1	hypothetical protein CG399_02610, partial (*Bifidobacteriaceae bacterium* NR015)
WP_106563713.1	ABC transporter permease (*Labedella gwakjiensis*)
GBU15610.1	integrase (*Polaromonas sp*.)
WP_066008415.1	tRNA (guanosine(46)-N7) -methyltransferase TrmB (*Campylobacter ornithocola*)
PYM16032.1	homoserine dehydrogenase (*Verrucomicrobia bacterium*)
WP_088892294.1	polysaccharide pyruvyl transferase family protein (*Leptolyngbya ohadii*)

**Table 4:** Proteins for which emPAI was significantly higher in the study group compared to the control group.

Bacteria Species	Domain	Phylum	Class	Order/Genus	Access Number
*Actinopolyspora erythraea*	*Bacteria*	*Actinomycetota*	*Actinomycetia*	*Actinosporaceae*	WP_043569372.1
*Listeria costaricensis*	*Firmicutes*	*Listeria*	*Bacilli*	*Listeriacea*	WP_0999222156.1
*Escherichia colli*	*proteobacteria*	*Pseudomonadot*	*Gammaproteobacteria*	*Enterobacteriacea*	AQU017941.1
*Methylobacterium* sp. *ap11*	*Bacteria*	*Methylobacterium*	*Aphaproteobacteria*	*Methylobacteriaceae*	WP_091753941.1
*Acidobacteria bacterium*	*Bacteria*	*Acidobacteriota*	*Acidobacteria*	*Acidobacteriales*	RMG44463.1
*candidate entotheonella* factor	*Bacteria*	*Tetomicrobia*		*entotheonella*	ETW97925.1
*Bacteroidet bacterium*	*Bacteria*	*Bacteroid*	*Saprospira*	*Bacteroides*	PIQ27132.1
*Paenisporasarcina* sp. *TG20*	*Bacteria*	*Paenisporosarcin*	*Bacilli*	*Planococcaceae*	WP_019414340.1
candidate *Thiodiazotropha endoloripes*	Nomenclatural status: not validly published				WP_0690002724.1
*Clostridiales bacterium*	*Bacteria*	*Eubacteriales*	*Clostrid*	*Clostridaceae*	PWM01737.1

**Table 5:** Proteins for which emPAI was significantly higher in the control group compared to the study group.

Bacteria Species	Domain	Phylum	Class	Order/Genus	Access Number
*Flavobacteriales bacterium 33_180_T64*	*Bacteria*	*Bacteroides*	*Flavobacteria*	*Flavobacteriales*	OUS03466.1
*Aureimonas* sp. *AU12*	*Bacteria*	*Pseudomonadot*	*Alphaproteo bacteria*	*Hyphomicrobiales*	WP_062208650.1
*Bacillus cerus*	*Bacteria*	*Bacillot*	*Bacilli*	*Bacillales*	WP_000124192.1

## Data Availability

The data presented in this study are available on request from the corresponding author.

## Supplementary Materials

The following supporting information can be downloaded at: https://www.mdpi.com/article/10.3390/ijms24086922/s1.

## Abbreviations

ADABA	circularization of gamma-N-acetyl-alpha: gamma-diaminobutyric acid
APGAR	appearance, pulse, grimace, activity, respiration score
BMI	body mass index
CRL	crown–rump length
CPR	cerebroplacental ratio
EFW	estimated fetal weight
emPAI	Exponentially Modified Protein Abundance Index
FGR	fetal growth restriction
HMP	Human Microbiome Project
NGS	next-generation sequencing
OPPsP	opportunistic premise plumbing pathogen
PE	preeclampsia
PI	pulsation index
RI	resistance index
TNF	tumor necrosis factor
TORCH	toxoplasmosis, other, rubella, cytomegalic and herpes infection
