# Supplementary material for: Comparative Analysis of the Placental Microbiome in Pregnancies with Late Fetal Growth Restriction versus Physiological Pregnancies
Source: Int J Mol Sci. 2023 Apr 7;24(8):6922. doi: 10.3390/ijms24086922 (PMC10139004; doi:10.3390/ijms24086922)
Supplement: Supplementary file 1 [file ijms-24-06922-s001.zip › ijms-2295832-supplementary.pdf]

Supplementary materials

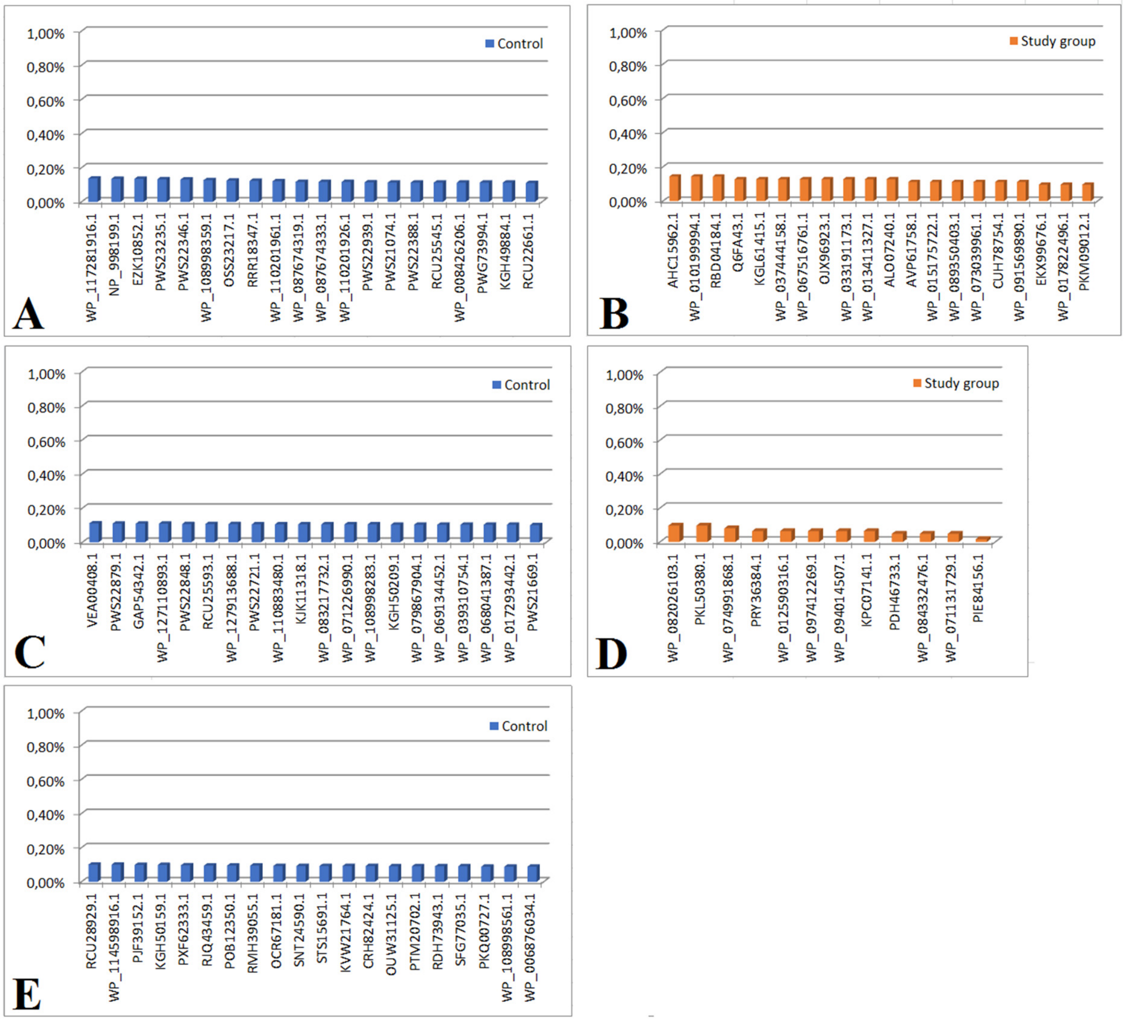

Supplementary Figure S1. Bar graphs showing the content of proteins (in order of decreasing emPAI value) that were present only in the material from the study group or control. Graph showing the content of proteins found only in the material from the control group: proteins 61-80 (**A**); proteins 81-100 (**C**); proteins 101–120 (**E**) or tested: proteins 61–80 (**B**); proteins 81-93 (**D**).

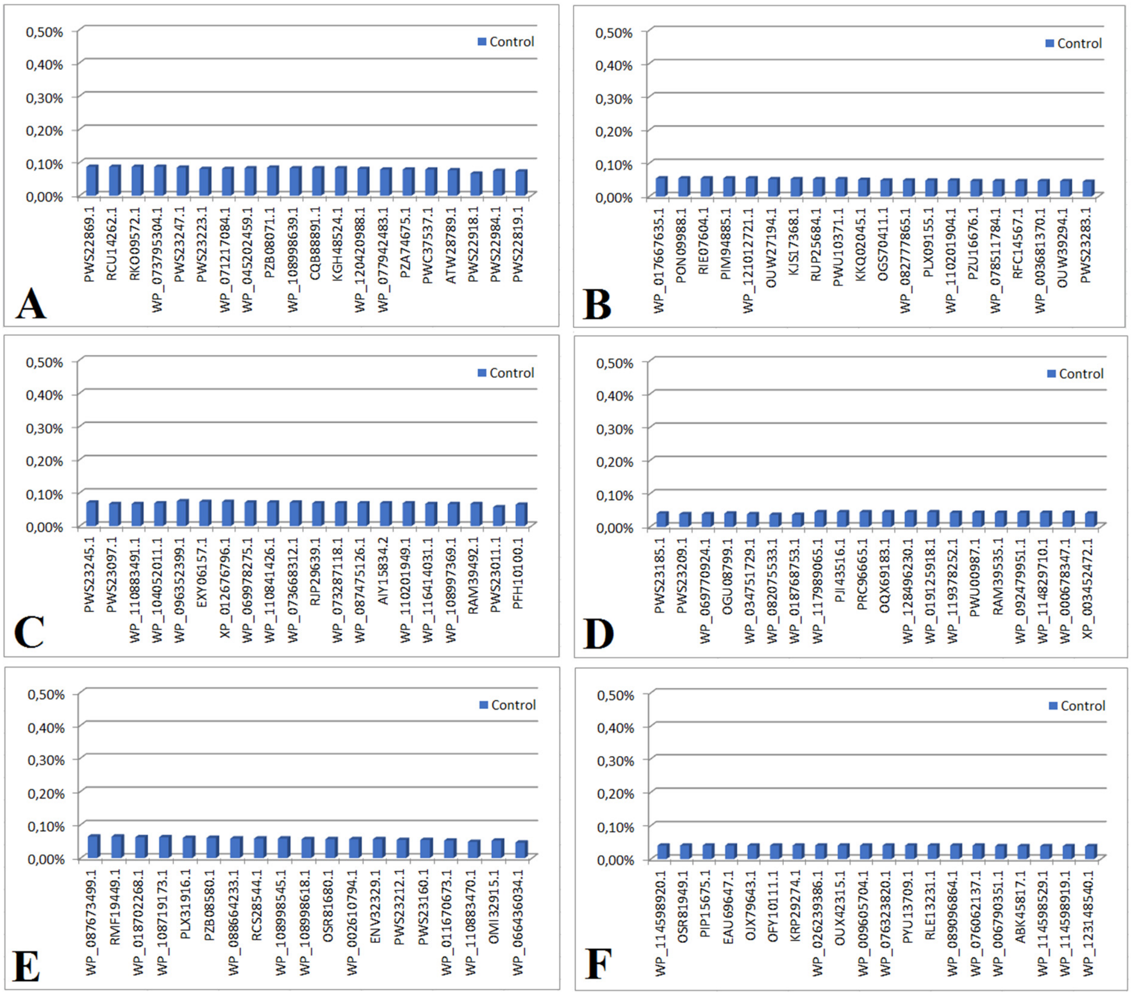

Supplementary Figure S2. Bar charts showing the content of proteins (in order of decreasing emPAI value) that were present only in the material from the control subjects. Proteins: 121-140 (**A**); 141-160 (**B**); 161-180 (**C**); 181-200 (**D**); 201-220 (**E**); 221-240 (**F**).

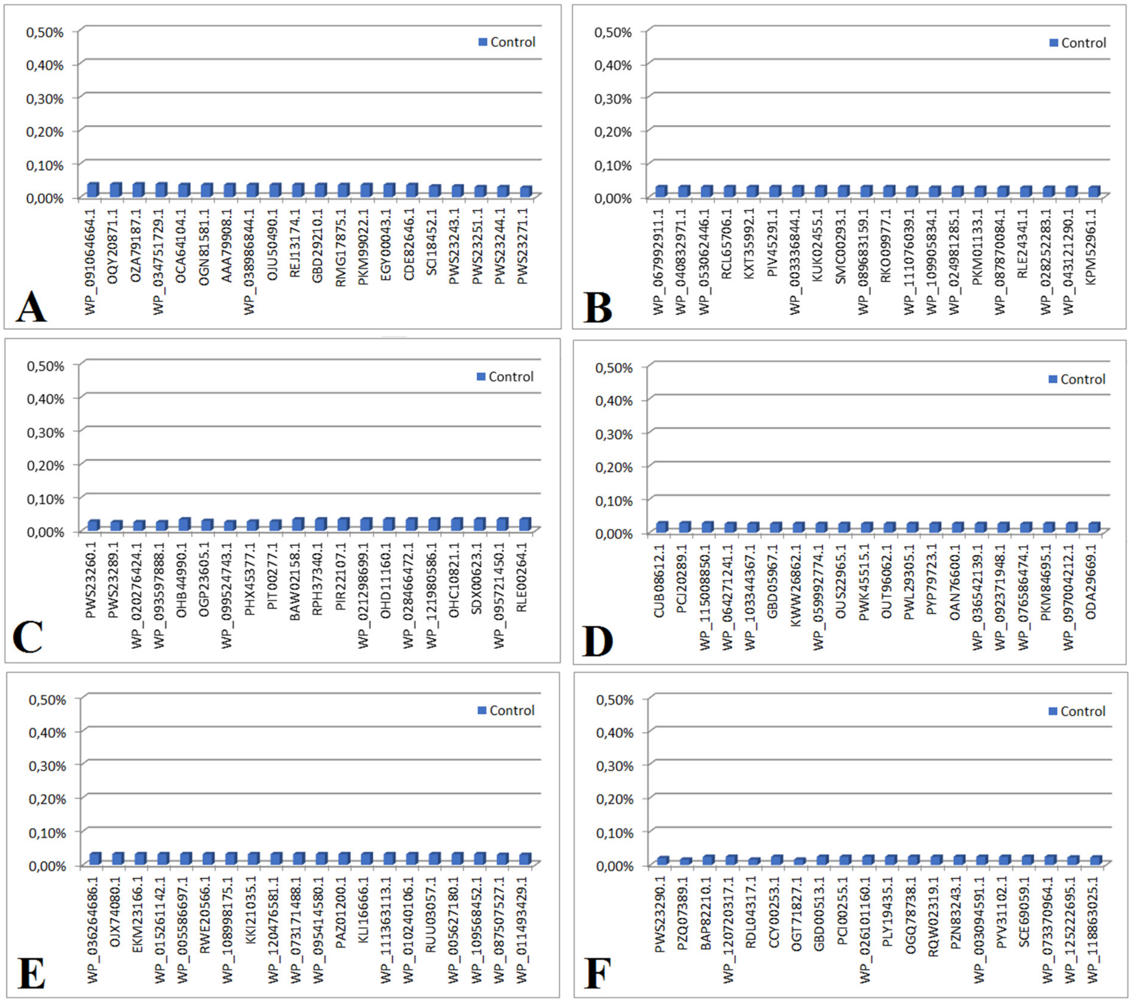

Supplementary Figure S3. Bar charts showing the content of proteins (in order of decreasing emPAI value) that were present only in the material from the control subjects. Proteins: 241-260 (**A**); 261-280 (**B**); 281-300 (**C**); 301-320 (**D**); 321-340 (**E**); 341-360 (**F**).

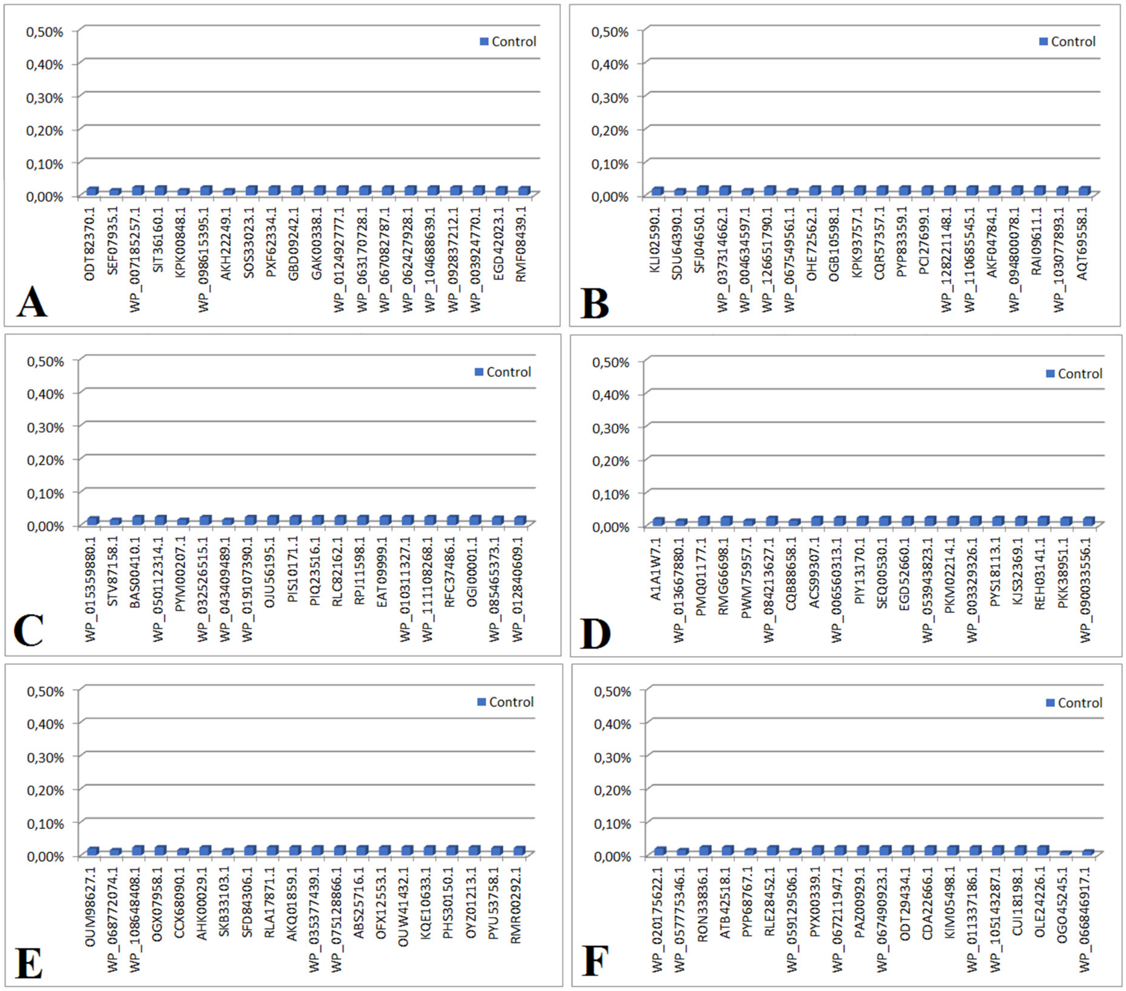

Supplementary Figure S4. Bar charts showing the content of proteins (in order of decreasing emPAI value) that were present only in the material from the control subjects. Proteins: 361-380 [**A**].381-400 (**B**) 401-420 (**C**). 421-440 (**D**). 441-460 (**E**). 461-480 (**F**).

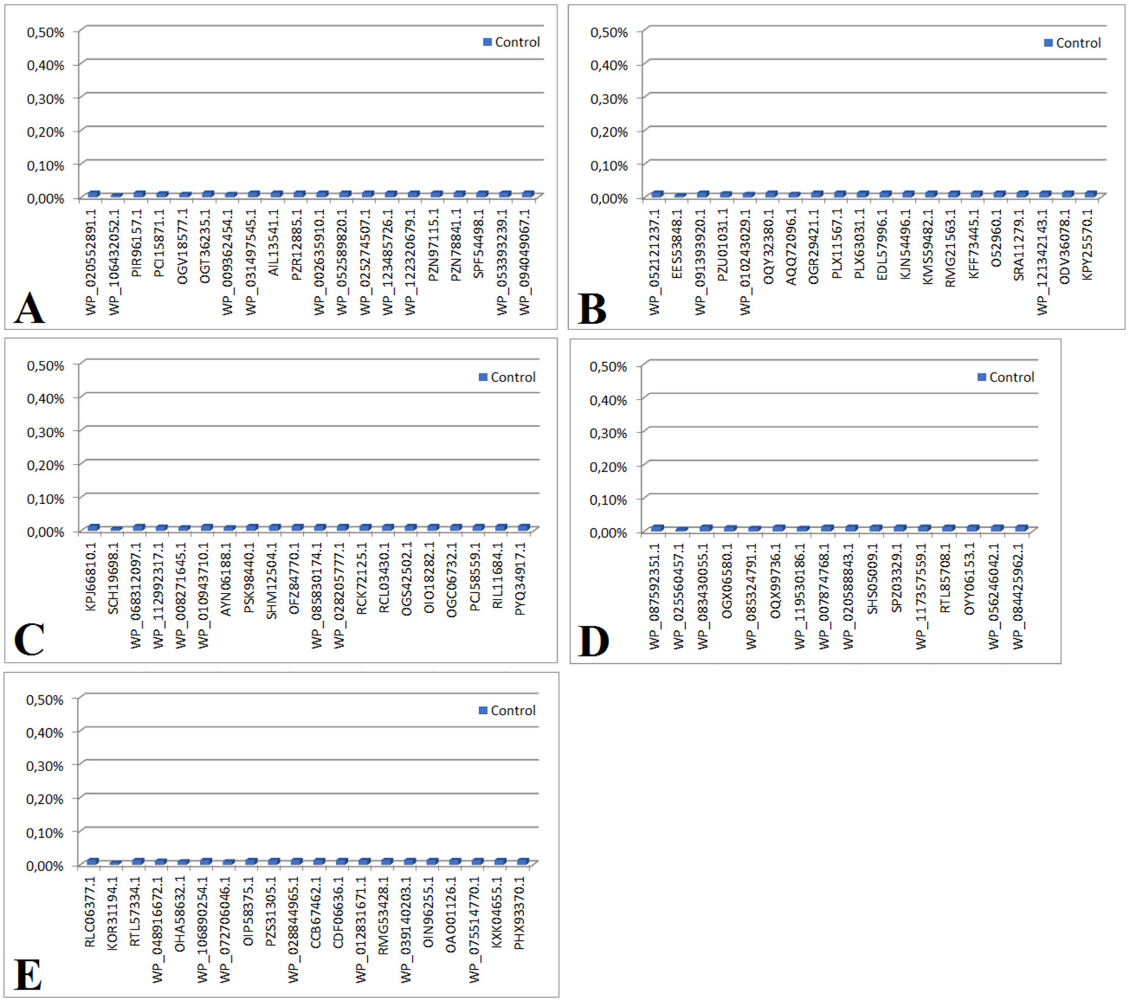

Supplementary Figure S5. Bar charts showing the content of proteins (in order of decreasing emPAI value) that were present only in the material from the control subjects. Proteins: 481-500 (**A**); 501-520 (**B**); 521-540 (**C**); 541-560 (**D**); 561-576 (**E**).
